# Morphology Tuning and Its Role in Optimization of Perovskite Films Fabricated from A Novel Nonhalide Lead Source

**DOI:** 10.1002/advs.202002296

**Published:** 2020-11-01

**Authors:** Jinwen Gu, Faming Li, Zenghui Wang, Yiran Xie, Lihe Yan, Peng Zeng, Hua Yu, Mingzhen Liu

**Affiliations:** ^1^ School of Materials and Energy University of Electronic Science and Technology of China Chengdu 611731 P. R. China; ^2^ Center for Applied Chemistry University of Electronic Science and Technology of China Chengdu 611731 P. R. China; ^3^ Key Laboratory for Physical Electronics and Devices of the Ministry of Education & Shaanxi Key Lab of Information Photonic Technique School of Electronics & Information Engineering Xi'an Jiaotong University Xi'an 710049 P. R. China; ^4^ Institute of Photovoltaics Southwest Petroleum University Chengdu 610500 P. R. China

**Keywords:** carrier lifetime, grain boundaries, lead formate, nonhalide lead source

## Abstract

Usage of nonhalide lead sources for fabricating perovskite solar cells (PSCs) has recently attracted increasing attention as a promising route toward realizing high quality PSC devices. However, the unique role of nonhalide lead sources in improving perovskite film morphology and PSC performance has largely remained unexplored, impeding broader application of these materials. Here, it is demonstrated that by using a new nonhalide lead source, lead formate (Pb(HCOO)_2_), good control of perovskite film morphology can be achieved. With the usage of lead formate, PbI_2_ can nicely border the perovskite grain boundaries (GBs) and form domain “walls” that segregate the individual perovskite crystal domains. The PbI_2_ at the GBs lead to significant improvement in film quality and device performance through passivating the defects at the perovskite GBs and suppressing lateral carrier diffusion. An impressive carrier lifetime at the microsecond scale (*τ*_2_ = 1714 ns) is achieved, further with an optimal power conversion efficiency of 20.3% for the resulting devices. This work demonstrates a promising and effective method toward fabricating high‐quality perovskites and high‐efficiency PSCs.

## Graphical abstract

A new nonhalide lead source, lead formate (Pb(HCOO)_2_), is used to prepare perovskite solar cells. Using Pb(HCOO)_2_ forms PbI_2_ domain "walls" bordering perovskite grains, leading to an ultralong carrier lifetime (≈1.7 microseconds) and a record‐high power conversion efficiency (>20%) for MAPbI_3_ based devices produced from nonhalide lead sources.

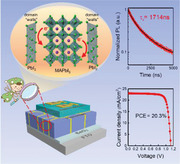

## 1 Introduction

Organic–inorganic lead halide perovskite solar cells (PSCs) have recently attracted significant attention due to their excellent photovoltaic performance. To date, the power conversion efficiency (PCE) of PSCs has soared to a verified value of 25.2%,^[^ ^1^ ^]^ rapidly approaching the Shockley–Queisser limit for single junction solar cells. Perovskite materials, constituting the key component in PSCs‐the light absorbing layer, possess superior optical and electronic properties ideally suited for photovoltaic devices, including high absorption coefficient,^[^ ^2^, ^3^ ^]^ high carrier mobility,^[^ ^4^, ^5^, ^6^ ^]^ long minority carrier lifetimes,^[^ ^7^, ^8^ ^]^ long charge diffusion lengths,^[^ ^8^ ^]^ and have been a key focus in PSC researches. Perovskite materials are generally characterized by their ABX_3_ structure. In organic–inorganic perovskites, typically A = organic cation (CH_3_NH_3_, NH_2_CHNH_2_, etc.), B = bivalent metal (Pb or Sn), and X = halide (Cl, Br, I, etc.).^[^ ^9^, ^10^, ^11^ ^]^ As an archetypical perovskite, CH_3_NH_3_PbI_3_ (methylammonium lead triiodide, MAPbI_3_) is renowned for its excellent photovoltaic performance, relatively simple crystal structure and facile production methods,^[^ ^12^, ^13^ ^]^ and has thus been the focus of PSC research from the beginning, while also providing the starting point to study its many derivatives (e.g., via doping) and variations (e.g., via substitution).^[^ ^14^, ^15^, ^16^, ^17^ ^]^

For synthesizing MAPbI_3_, typical process involves dissolving lead halide source (e.g., PbI_2_) and alkyl ammonium halide in an organic solvent.^[^ ^5^, ^18^, ^19^, ^20^ ^]^ Recently, usage of nonhalide lead sources has attracted increasing attention^[^ ^21^, ^22^, ^23^, ^24^, ^25^ ^]^ due to resultant improvements in fabrication process and film quality, such as fast crystallization at low temperature,^[^ ^23^ ^]^ superior stability,^[^ ^22^ ^]^ and ultrasmooth surface,^[^ ^25^ ^]^ all of which are key toward successful manufacturing of commercial‐grade PSC products. Although nonhalide lead sources have been investigated for a few years, the correlation between optoelectronic properties of these films and the corresponding device performance are still lack of understanding. Toward its broad adoption, a number of important issues remain yet to be addressed. For example, study on the effects of morphology tuning for PSCs derived from nonhalide lead sources remains elusive. In particular, it has been known that grain boundaries (GBs) plays a vital role in PSC performance as it determines a series of key factors, such as carrier lifetime,^[^ ^24^ ^]^ trap density,^[^ ^26^ ^]^ current hysteresis,^[^ ^25^, ^27^ ^]^ and photovoltaic (PV) performance.^[^ ^25^, ^28^, ^29^ ^]^ While a number of methods have been proposed to suppress the defects at the perovskite GBs, such as Lewis bases treatment,^[^ ^30^ ^]^ adding fullerene,^[^ ^27^ ^]^ constructing “patches,”^[^ ^31^ ^]^ introduction of CH_3_NH_3_I (MAI) healing layer,^[^ ^32^ ^]^ or PbI_2_ materials,^[^ ^33^ ^]^ such effort has been limited to perovskite derived from conventional halide lead sources. Further, a systematic understanding has remained elusive for the functions of the nonhalide lead sources in the film morphology and optoelectronic response of PSC devices, such as their effect on the defect states and carrier transport in the resulting perovskite film. Hence, it is of both practical and fundamental importance to explore novel strategies that can both leverage the benefits associated with the usage of nonhalide lead sources and achieve PSCs with high PCE values, while performing systematic characterization on the film formation and carrier dynamics toward better understanding of the underlying mechanisms.

Here we demonstrate a facile solution process for fabricating high‐quality, high‐efficiency PSCs based on a new nonhalide lead source, Pb(HCOO)_2_ (lead formate), and systematically study the effects of its morphological controlling. In particular, we focus on the formation and role of PbI_2_ at the GBs in such perovskite films. We find that the growth of PbI_2_ crystals can be controlled to embed in the GBs by tuning the ratio between Pb(HCOO)_2_ and MAI, and that PbI_2_ can effective form domain “walls” at GBs between individual perovskite crystal domains and help passivate defect states, thereby improving the performance of perovskites. Benefitting from the suppressed lateral carriers diffusion from the “walls,” the perovskite films exhibit an impressive minority carrier lifetime on the microsecond scale (*τ*_2_ = 1714 ns), rivaling MAPbI_3_ single crystal.^[^ ^4^, ^34^, ^35^ ^]^ Using this nonhalide lead source, we achieve an optimized PSC with a PCE value of 20.3%. Furthermore, through systematic comparison of perovskites formed from different lead sources, we find that the usage of lead formate shows significant advantages in controlling PbI_2_ walls at GBs and leading to superior device performance. Our findings can help pave the pathways toward facile production of high‐quality PSCs, and advance the understanding of film formation that can be further leveraged to enhance the device performance.

## 2 Results and Discussion

### 2.1 Fabrication Process of the Perovskite Films

**Figure 1:**
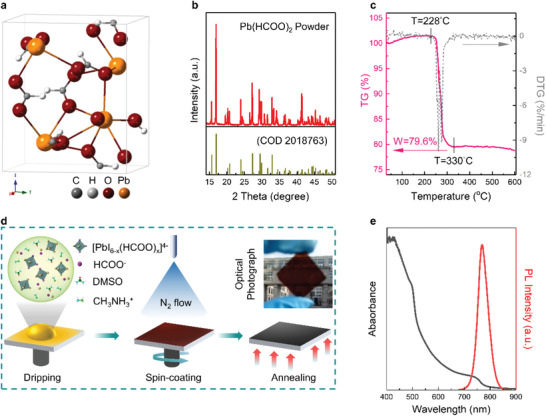
Structure and physical properties of non‐halide lead salt, Pb(HCOO)_2_. a) Crystal structure of Pb(HCOO)_2_. b) XRD patterns of Pb(HCOO)_2_ powder. c) TG‐differential TG curve of Pb(HCOO)_2_ powder. d) Schematic diagram of fabrication process for the perovskite films via Pb(HCOO)_2_ route. e) Absorption and PL spectra for the MAPbI_3_ perovskite films fabricated by the Pb(HCOO)_2_ routes.

We employ a nonhalide lead source, Pb(HCOO)_2_, with orthorhombic crystal structure (space group: *Pnma*)^[^ ^36^ ^]^ in fabricating perovskite films (**Figure** **1**a). The X‐ray diffraction (XRD) of the Pb(HCOO)_2_ powder well matches that from the Crystallography Open Database (COD 2 018 763) (Figure 1b). Thermogravimetry (TG) analysis (Figure 1c) confirms that the Pb(HCOO)_2_ powder shows sudden and steep weight loss at from 228 to 330 °C (generation of PbO), and remains 79.6% of original weight at 330 °C, further confirming the composition of the Pb(HCOO)_2_ source.^[^ ^37^ ^]^ The perovskite films are prepared by dissolving the nonhalide lead source, Pb(HCOO)_2_, and the alkyl ammonium halide source, CH_3_NH_3_I (MAI) in organic solvent, such as *N*,*N*‐dimethylformamide (DMF) (Figure S1, Supporting Information). We optimize the process by varying a number of conditions, including adding dimethyl sulphoxide (DMSO) in the precursor (to control the crystal growth rate),^[^ ^38^ ^]^ introducing nitrogen flow during spinning (to adjust the nucleation density),^[^ ^39^ ^]^ and using thermal annealing as a post processing (to tune the crystallization of the perovskite films). Upon examining the resultant films using scanning electron microscope (SEM), Fourier‐transform infrared spectroscopy, and XRD (Figures S2–S5, Supporting Information), we determine that the optimal processing condition involves applying 10% DMSO additive into the solution followed by nitrogen flowing and thermal annealing. The resultant films exhibit typical absorption and emission spectra for high‐quality perovskites (Figure 1e).^[^ ^40^, ^41^ ^]^

### 2.2 GBs and Its Forming Mechanism

**Figure 2:**
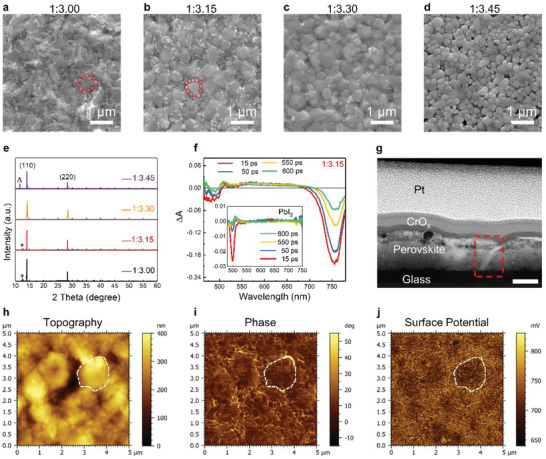
Structure and morphology of perovskite films. a–d) Top‐view SEM images of perovskite films via Pb(HCOO)_2_ route, with various ratios of Pb(HCOO)_2_ to MAI in the precursor. a) 1:3.00, b) 1:3.15, c) 1:3.30, d) 1:3.45. e) XRD patterns of MAPbI_3_ perovskite films with various ratios of Pb(HCOO)_2_/MAI in the precursors. The characteristic peaks of PbI_2_ and MAI are marked with asterisk and triangle, respectively. f) fs‐TA spectra of perovskite film at various delays after photoexcitation, the perovskite film with the ratio of Pb(HCOO)_2_:MAI = 1:3.15. Inset shows the TA spectra of pure PbI_2_ crystals. g) The cross‐sectional TEM image of sample with a device structure of glass/perovskite/CrO*_x_*/Pt. Scale bar at 200 nm. h–j) KPFM measurements performed on a perovskite/TiO_2_/FTO glass structure. h) Topography image, i) phase, and j) CPD mapping of perovskite film with the ratio of Pb(HCOO)_2_:MAI = 1:3.15.

In order to gain insight into the role that Pb(HCOO)_2_ plays in governing the morphological properties of perovskite films, we systematically vary the molar ratio between Pb(HCOO)_2_ and MAI in the DMF solution (1:3.00, 1:3.15, 1:3.30, and 1:3.45). SEM images show that PbI_2_ can form at the GBs (bright particles in SEM images,^[^ ^42^, ^43^ ^]^ outlined by red circles in **Figure** **2**a,b), and its formation can be effectively tuned by adjusting the starting materials. The density of PbI_2_ decreases as the amount of MAI increases (Figure 2a–d), and completely disappears at 1:3.45 (Figure 2d), consistent with the known fact that residual PbI_2_ can easily react with excessive MAI. In particular, at the ratio of 1:3.15, PbI_2_ is mostly formed at the GBs between perovskite crystalline domains, demonstrating good control over its density and morphology. XRD results (Figure 2e) confirm this trend in the evolution of the diffraction peak of PbI_2_ crystal at 2*θ* = 12.6° (Figure S6, Supporting Information), and show that the perovskite films are highly crystalized in the (110) and (220) directions. Femtosecond Transient Absorption (fs‐TA) spectroscopy further confirms the above observation: at the ratio of 1:3.15, the PbI_2_ feature (bleaching at 490 nm and induced absorption at 520 nm, Figure 2f inset)^[^ ^44^, ^45^ ^]^ is present in the TA spectrum; while at higher MAI ratio, the PbI_2_ feature vanishes, and only the band‐edge transitions in the perovskite (strong bleaching at 760 nm) remains visible (Figure S7, Supporting Information). Importantly, we find that PbI_2_ forms not only on the film surface, but also continue through the entire film thickness, as confirmed by the cross‐sectional transmission electron microscope (TEM) and SEM images (Figure 2g; and Figure S8, Supporting Information). We therefore conduct scanning Kelvin probe microscopy (KPFM) measurement for the 1:3.15 film (Figure 2h–j). Contact potential difference (CPD) mapping reveals that the GBs have higher surface potential (Figure 2j), which is attributed to the difference in work function of PbI_2_ and perovskite,^[^ ^33^ ^]^ showing the presentence of domain walls formed by PbI_2_.

All the above observations show that using Pb(HCOO)_2_ and MAI with carefully‐tuned ratio as the starting materials can lead to controllable formation of PbI_2_ crystals at the perovskite GBs. It is important to note that this is only observed for perovskite films made from Pb(HCOO)_2_/MAI; other organic acid‐based lead sources, such as lead acetate ((PbCH_3_COO)_2_, i.e., Pb(Ac)_2_), has less control over the formation of PbI_2_ at the GBs (Figure S9a–d, Supporting Information).

**Figure 3:**
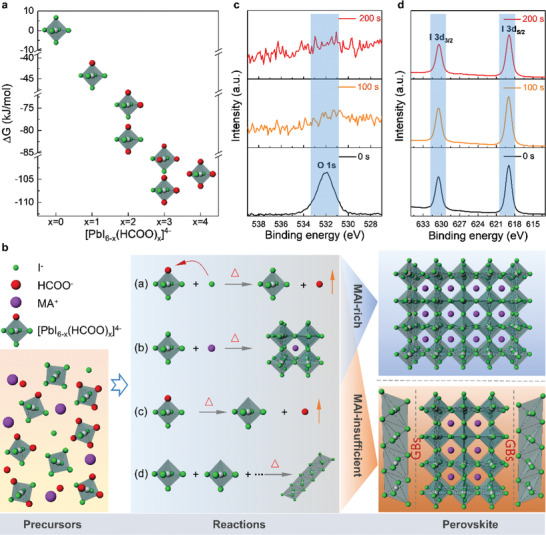
Formation mechanism of perovskite films via Pb(HCOO)_2_ route. a) Calculated values of △G versus the different octahedral structure, [PbI_6−_ *_x_*(HCOO)*_x_*]^4−^ (0≤*x*≤4). b) Schematic representation of the main stages that transition from precursor to perovskite. (For the [PbI_6−_ *_x_*(HCOO)*_x_*]^4−^ (1≤*x*≤4) octahedral, take *x* = 1 as example). c,d) XPS spectra of perovskite films via Pb(HCOO)_2_ route, the sample was etched using the Ar^+^ plasma for 0s, 100s, 200s. Narrow scanning XPS spectra of O 1s c) and I 3d d).

To understand the unique role of HCOO^−^ in the formation of perovskite films, we employ density functional theory to evaluate the Gibbs free energy (△G) of all possible octahedral structures [PbI_6−_ *_x_*(HCOO)*_x_*]^4−^ (0≤*x*≤6) in the precursor (**Figure** **3**a).^[^ ^46^ ^]^ We find △G < 0 for structures with *x* ≤ 4, suggesting they can be spontaneously formed in perovskite precursors.^[^ ^22^ ^]^ In contrast, structures with *x* = 5,6 do not converge in calculation and thus cannot form.^[^ ^47^ ^]^ These observations are further confirmed by the absorption spectra of the precursor (Figure S10, Supporting Information), and consistent with observation in other nonhalide lead sources.^[^ ^22^, ^48^ ^]^

When MAI and Pb(HCOO)_2_ react in the precursor (Figure 3b), [PbI_6_]^4−^ (*x* = 0) octahedral first self‐assemble with MA^+^ and crystalize into MAPbI_3_ (reaction b). Other octahedral (1≤*x*≤4) also gradually convert to [PbI_6_]^4−^ through anion exchange (HCOO^−^ to I^−^, reaction a)^[^ ^49^ ^]^ before forming perovskites, while the remaining HCOO^−^ is vaporized during heating. Such delayed process could contribute to better crystallization of the film. Interestingly, new reaction product arises when there is insufficient MAI: the I^−^ deficiencies lead to incomplete exchange of HCOO^−^ (reaction c), thus cause point sharing [PbI_6−_ *_x_*(HCOO)*_x_*]^4−^ octahedral to transform into face sharing [PbI_6_]^4−^ octahedral, eventually forming PbI_2_ upon loss of MA^+^ (reaction d) at the perovskite GBs, as the sample shown in Figure 2b.

To confirm the absence of HCOO^−^ in the resulting films, we use X‐ray photoelectron spectroscopy (XPS) to examine the presence of oxygen (i.e., O 1s) and iodine (i.e., I 3d) inside films etched by Ar^+^ plasma (Figure 3c,d). The results confirm that oxygen signal only exists on the surface of perovskite, likely due to surface adsorbates (Figure S11, Supporting Information).^[^ ^50^, ^51^ ^]^ The complete absence of O element inside the perovskite confirms that no HCOO^−^ is left in the final product, consistent with the picture that all [PbI_6−_ *_x_*(HCOO)*_x_*]^4−^ loose HCOO^−^ through ion exchange before forming perovskite crystal and GBs. It further confirms that the GBs do not contain any HCOO^−^ or PbO. With good understanding of the formation of PbI_2_ at the GBs, we now focus on systematically analyzing their effects on passivating the defect states and improving device performance.

### 2.3 Tuning of Carrier Dynamics

**Figure 4:**
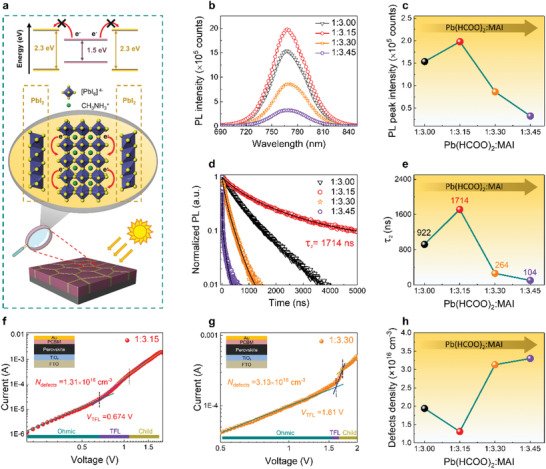
Tuning of carrier dynamics and defect states. a) Schematic illustration of PbI_2_ orderly bordering at the GBs of MAPbI_3_ perovskite films. b,c) The steady‐state PL spectra for the MAPbI_3_ perovskite films fabricated from the different molar ratios of Pb(HCOO)_2_/MAI b), PL peak intensity as a function of the ratio of Pb(HCOO)_2_/MAI c). d,e) The TRPL spectra for the MAPbI_3_ perovskite films fabricated with different molar ratios of Pb(HCOO)_2_/MAI, the excitation wavelength@510 nm d). Carrier lifetime as a function of the ratio of Pb(HCOO)_2_/MAI e). f–h) Current–voltage curve for an electron‐only device (structure: Au/PCBM/Perovksite/TiO_2_/FTO glass) based on perovskite films with the ratio of Pb(HCOO)_2_:MAI = 1:3.15 f), and 1:3.30 g). The trap density derived from current–voltage curve with different ratios of Pb(HCOO)_2_/MAI h).

To obtain more insight into the role of Pb(HCOO)_2_ in enhancing the optoelectronic properties of perovskite films, and to evaluate carrier diffusion kinetics properties of perovskite films with PbI_2_ walls at GBs, we perform both steady‐state photoluminescence (PL) and time‐resolved PL (TRPL) measurements (**Figure** **4**b,d) on the films prepared with varying ratio of Pb(HCOO)_2_:MAI. All samples exhibit PL features at 768 nm, consistent with expected optical properties of MAPbI_3_ films (Figure 4b). Importantly, the measured PL intensity show clear correlation with the morphology of the perovskite films (Figures 4c, and 2a–d), with peak PL value found at the ratio of Pb(HCOO)_2_:MAI = 1:3.15, in which the perovskite crystalline domains are nicely bordered by PbI_2_ (Figure 2b; and Figure S12, Supporting Information). Furthermore, TRPL measurements (Figure 4d) show that carrier lifetime follows the exactly same trend (Figure 4e), with the film prepared with Pb(HCOO)_2_:MAI = 1:3.15 exhibiting an impressively long carrier lifetime (*τ*) in the microsecond range (Table S1, Supporting Information), *τ*_2_ = 1714 ns (with A_2_ = 61%; *τ*_1_ = 460 ns, A_1_ = 39%; *τ*_avg_ = 1224 ns). This remarkably long carrier lifetime of our sample is even tantamount to that of most MAPbI_3_ single crystals.^[^ ^4^, ^34^, ^35^ ^]^ We note that the usage of Pb(HCOO)_2_ is advantageous for achieving such long lifetime; as a comparison, perovskite made from Pb(Ac)_2_ produces a much shorter lifetime (*τ*_2_ = 437 ns, shorter by one order of magnitude; see Figure S9e–f and Table S2, Supporting Information). Here we again find clear correlation between carrier lifetime and film morphology. Specifically, even in the Pb(Ac)_2_ derived films with longest lifetime (1:3.15 ratio), the PbI_2_ only loosely decorates the perovskite GBs (Figure S9b, Supporting Information), while in the films made from Pb(HCOO)_2_ the PbI_2_ form a complete boundary around the perovskite crystalline domains (Figure 2b). Besides, TRPL decay measurements provide a direct insight into the trap‐state induced carrier dynamics. We applied a simulation model based on our previous work^[^ ^52^ ^]^ to build a clear connection between defect recombination and carrier lifetime (Figure S13 and Table S3, Supporting Information) and indicate that the reduced defect density in 1:3.15 sample leads to its increased carrier lifetime. This suggests that by carefully choosing and tuning the starting materials, PbI_2_ can effective form domain “walls” that segregate the individual perovskite crystal domains, thus effectively passivating the defects at the perovskite GBs and suppressing lateral diffusion and recombination of the carriers (Figure 4a).^[^ ^33^ ^]^

To further evaluate such passivation effects, we quantify the density of defects in these MAPbI_3_ films using space‐charge‐limited current (SCLC) measurements. We fabricate electron‐only devices with the structure of fluorine‐doped tin‐oxide(FTO)/TiO_2_/MAPbI_3_/[6,6]‐phenyl‐C61‐butyric acid methyl ester (PCBM)/Au and characterize their dark current–voltage response (Figure 4f–g; and Figure S14, Supporting Information). The trap states density (*N*_defects_) is estimated by^[^ ^4^ ^]^

(1) $$\begin{array}{c} N_{\mathrm{defects}}=\frac{2\varepsilon \varepsilon_{0}}{eL^{2}}\;V_{\mathrm{TFL}} \end{array}$$

where *V*_TFL_ represents the trap‐filled limit voltage, *e* is the electronic charge, *L* is the thickness of the perovskite film, *ε* is the relative dielectric constant of MAPbI_3_ (*ε = 32*),^[^ ^53^ ^]^ and *ε*_0_ is the vacuum permittivity. As expected, lower defect density corresponds to longer carrier lifetime, with the best value (1.31 × 10^16^ cm^−3^) achieved again for the Pb(HCOO)_2_:MAI = 1:3.15 film (Figure 4h), in which the PbI_2_ nicely filling the perovskite GBs (Figure 2b). These results confirm that the trap densities can be significantly reduced by controlling the density and morphology of PbI_2_ crystal at perovskite GBs, which lead to long carrier lifetimes.

### 2.4 Photovoltaic Performance

**Figure 5:**
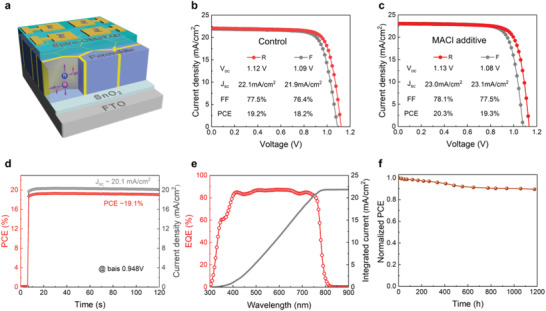
PV performance of PSCs based on the Pb(HCOO)_2_ source. a) Schematic illustration of device architecture, with a device structure of glass/FTO/SnO_2_ compact layer/perovskite/HTM/Au. b–c) *J–V* curve for the champion device (molar ratio of Pb(HCOO)_2_:MAI = 1: 3.15) in reverse (R) and forward (F) scan directions. The devices based on perovskite films without b) and with MACl additive c). d) Steady‐state PCE and current density of the champion device with MACl additive measured under a bias@0.948 V. e) EQE spectrum and the integrated current density of the champion device with MACl additive. f) Long‐term stability of the devices stored in a dry box (temperature ≈25 °C and RH≈10%).

To confirm that the optimized embedded PbI_2_ crystals at the GBs and long carrier lifetime can help achieve better PCE, we assess the photovoltaic performance of resulting PSCs (**Figure** **5**a). As expected, a similar trend (PCE dependence on the molar ratio) is observed (Figure S15, Supporting Information), with the best‐performing devices achieved for films made with the molar ratio of 1: 3.15. The champion device exhibits an optimized PCE of 19.2%, yielding short‐circuit current density (*J*_sc_), open‐circuit voltage (*V*_oc_), and fill factor (*FF*) of 22.1 mA cm^−2^, 1.12 V and 77.5%, respectively (Figure 5b). We also show that the PCE can be further enhanced through simple additive engineering which has been known as an effectively way to enhance PCE in MAPbI_3_ PSCs.^[^ ^54^, ^55^ ^]^ As a demonstration, we introduce MACl as additive in the precursor to assist the crystal growth (see the experimental procedures for details).^[^ ^56^ ^]^ The resulting devices show clearly improved PCE values up to 20.3% (Figure 5c), by far surpassing all PSCs made from pure nonhalide sources (Tables S4 and S5, Supporting Information), with a stabilized value of 19.1% (Figure 5d). We also measure its external quantum efficiency (EQE), from which we obtain an integrated *J*_sc_ value of 21.9 mA cm^−2^_ _(Figure 5e), in good agreement with the *J–V* scan result (Figure 5c). The slight mismatch is likely due to the spectral mismatch between the light sources of the EQE and solar simulator, as commonly found in literature.^[^ ^55^, ^57^ ^]^ In addition, the device exhibits remarkable stability, with measured PCE retaining at 89% of initial value after 1000 h without any encapsulation (Figure 5f). The testing of photo and humidity stability further demonstrated that devices with optimized amount of PbI_2_ GBs (1:3.15) show obvious better stability than those with minor PbI_2_ GBs (1:3.30) under all conditions (Figure S16, Supporting Information), certifying the role of these GBs in protecting perovskite grains. The above examples demonstrate that by using Pb(HCOO)_2_ as the starting material, and by carefully controlling the molar ratio, one can achieve optimized film morphology with PbI_2_ filling the perovskite GBs leading to high‐quality PSCs.

## 3 Conclusion

In conclusion, we report a new nonhalide lead source, lead formate Pb(HCOO)_2_, for fabricating high‐quality perovskite films, which offers a unique pathway for controlling the decoration of PbI_2_ at the perovskite GBs. In perovskite films fabricated with the optimized Pb(HCOO)_2_:MAI ratio, the PbI_2_ crystals filling the MAPbI_3_ GBs can effectively passivate the defects, consistent with the SCLC measurements, and lead to a significant enhancement in the carrier lifetime, reaching an impressively value of *τ*_2_ = 1714 ns. This allows us to achieve PSCs with an optimal PCE of 20.3%, surpassing all PSCs made from nonhalide sources. Our work demonstrates a strategy for optimizing device performance through careful control of film morphology, revealing great potentials of Pb(HCOO)_2_ as the starting material for pushing PSCs into industrial application due to its low cost and facile fabrication method while retaining the superior performance of devices.

## Conflict of Interest

The authors declare no conflict of interest.

## Supporting information

Supporting information

## References

[1] Best Research‐Cell Efficiency Chart National Renewable Energy Laboratory, https://www.nrel.gov/pv/cell-efficiency.html (accessed: May 2020).

[2] B. R. Sutherland , S. Hoogland , M. M. Adachi , P. Kanjanaboos , C. T. Wong , J. J. McDowell , J. Xu , O. Voznyy , Z. Ning , A. J. Houtepen , E. H. Sargent , Adv. Mater. 2015, 27, 53. 25359103 10.1002/adma.201403965

[3] Y. Wang , S. Bai , L. Cheng , N. Wang , J. Wang , F. Gao , W. Huang , Adv. Mater. 2016, 28, 4532. 26669326 10.1002/adma.201504260

[4] D. Shi , V. Adinolfi , R. Comin , M. Yuan , E. Alarousu , A. Buin , Y. Chen , S. Hoogland , A. Rothenberger , K. Katsiev , Y. Losovyj , X. Zhang , P. A. Dowben , O. F. Mohammed , E. H. Sargent , O. M. Bakr , Science 2015, 347, 519. 25635092 10.1126/science.aaa2725

[5] W. Y. Nie , H. H. Tsai , R. Asadpour , J. C. Blancon , A. J. Neukirch , G. Gupta , J. J. Crochet , M. Chhowalla , S. Tretiak , M. A. Alam , H. L. Wang , A. D. Mohite , Science 2015, 347, 522. 25635093 10.1126/science.aaa0472

[6] Y. Liu , Z. Yang , D. Cui , X. Ren , J. Sun , X. Liu , J. Zhang , Q. Wei , H. Fan , F. Yu , X. Zhang , C. Zhao , S. Liu , Adv. Mater. 2015, 27, 5176. 26247401 10.1002/adma.201502597

[7] D. W. deQuilettes , S. M. Vorpahl , S. D. Stranks , H. Nagaoka , G. E. Eperon , M. E. Ziffer , H. J. Snaith , D. S. Ginger , Science 2015, 348, 683. 25931446 10.1126/science.aaa5333

[8] S. D. Stranks , G. E. Eperon , G. Grancini , C. Menelaou , M. J. Alcocer , T. Leijtens , L. M. Herz , A. Petrozza , H. J. Snaith , Science 2013, 342, 341. 24136964 10.1126/science.1243982

[9] F. Li , Y. Pei , F. Xiao , T. Zeng , Z. Yang , J. Xu , J. Sun , B. Peng , M. Liu , Nanoscale 2018, 10, 6318. 29589862 10.1039/c8nr00758f

[10] F. Li , M. Liu , J. Mater. Chem. A 2017, 5, 15447.

[11] B. R. Sutherland , E. H. Sargent , Nat. Photonics 2016, 10, 295.

[12] W. Chen , Y. Wu , Y. Yue , J. Liu , W. Zhang , X. Yang , H. Chen , E. Bi , I. Ashraful , M. Graetzel , L. Han , Science 2015, 350, 944. 26516198 10.1126/science.aad1015

[13] M. Liu , M. B. Johnston , H. J. Snaith , Nature 2013, 501, 395. 24025775 10.1038/nature12509

[14] S. H. Turren‐Cruz , A. Hagfeldt , M. Saliba , Science 2018, 362, 449. 30309904 10.1126/science.aat3583

[15] M. Saliba , T. Matsui , K. Domanski , J. Y. Seo , A. Ummadisingu , S. M. Zakeeruddin , J. P. Correa‐Baena , W. R. Tress , A. Abate , A. Hagfeldt , M. Gratzel , Science 2016, 354, 206. 27708053 10.1126/science.aah5557

[16] S. Ahmad , P. Fu , S. Yu , Q. Yang , X. Liu , X. Wang , X. Wang , X. Guo , C. Li , Joule 2019, 3, 794.

[17] J. Werner , G. Nogay , F. Sahli , T. C.‐J. Yang , M. Bräuninger , G. Christmann , A. Walter , B. A. Kamino , P. Fiala , P. Löper , S. Nicolay , Q. Jeangros , B. Niesen , C. Ballif , ACS Energy Lett. 2018, 3, 742.

[18] W. S. Yang , J. H. Noh , N. J. Jeon , Y. C. Kim , S. Ryu , J. Seo , S. I. Seok , Science 2015, 348, 1234. 25999372 10.1126/science.aaa9272

[19] X. Li , D. Bi , C. Yi , J.‐D. Decoppet , J. Luo , S. M. Zakeeruddin , A. Hagfeldt , M. Gratzel , Science 2016, 353, 58. 27284168 10.1126/science.aaf8060

[20] Y. Zong , Y. Zhou , Y. Zhang , Z. Li , L. Zhang , M.‐G. Ju , M. Chen , S. Pang , X. C. Zeng , N. P. Padture , Chem 2018, 4, 1404.

[21] D. T. Moore , H. Sai , K. W. Tan , D. M. Smilgies , W. Zhang , H. J. Snaith , U. Wiesner , L. A. Estroff , J. Am. Chem. Soc. 2015, 137, 2350. 25625616 10.1021/ja512117e

[22] A. M. Ganose , C. N. Savory , D. O. Scanlon , J. Phys. Chem. Lett. 2015, 6, 4594. 26525942 10.1021/acs.jpclett.5b02177

[23] D. T. Moore , H. Sai , K. Wee Tan , L. A. Estroff , U. Wiesner , Apl. Mater. 2014, 2, 081802.

[24] L. Zhao , D. Luo , J. Wu , Q. Hu , W. Zhang , K. Chen , T. Liu , Y. Liu , Y. Zhang , F. Liu , T. P. Russell , H. J. Snaith , R. Zhu , Q. Gong , Adv. Funct. Mater. 2016, 26, 3508.

[25] W. Zhang , M. Saliba , D. T. Moore , S. K. Pathak , M. T. Horantner , T. Stergiopoulos , S. D. Stranks , G. E. Eperon , J. A. Alexander‐Webber , A. Abate , A. Sadhanala , S. H. Yao , Y. L. Chen , R. H. Friend , L. A. Estroff , U. Wiesner , H. J. Snaith , Nat. Commun. 2015, 6, 6142. 25635571 10.1038/ncomms7142

[26] W. Zhang , S. Pathak , N. Sakai , T. Stergiopoulos , P. K. Nayak , N. K. Noel , A. A. Haghighirad , V. M. Burlakov , D. W. deQuilettes , A. Sadhanala , W. Li , L. Wang , D. S. Ginger , R. H. Friend , H. J. Snaith , Nat. Commun. 2015, 6, 10030. 26615763 10.1038/ncomms10030 PMC4674686

[27] Y. Shao , Z. Xiao , C. Bi , Y. Yuan , J. Huang , Nat. Commun. 2014, 5, 5784. 25503258 10.1038/ncomms6784

[28] J. Qing , H. T. Chandran , Y. H. Cheng , X. K. Liu , H. W. Li , S. W. Tsang , M. F. Lo , C. S. Lee , ACS Appl. Mater. Interfaces 2015, 7, 23110. 26442432 10.1021/acsami.5b06819

[29] T. S. Sherkar , C. Momblona , L. Gil‐Escrig , J. Avila , M. Sessolo , H. J. Bolink , L. J. A. Koster , ACS Energy Lett. 2017, 2, 1214. 28540366 10.1021/acsenergylett.7b00236 PMC5438194

[30] N. K. Noel , A. Abate , S. D. Stranks , E. S. Parrott , V. M. Burlakov , A. Goriely , H. J. Snaith , ACS Nano 2014, 8, 9815. 25171692 10.1021/nn5036476

[31] L. Liu , S. Huang , Y. Lu , P. Liu , Y. Zhao , C. Shi , S. Zhang , J. Wu , H. Zhong , M. Sui , H. Zhou , H. Jin , Y. Li , Q. Chen , Adv. Mater. 2018, 30, 1800544. 10.1002/adma.201800544 29882254

[32] D.‐Y. Son , J.‐W. Lee , Y. J. Choi , I.‐H. Jang , S. Lee , P. J. Yoo , H. Shin , N. Ahn , M. Choi , D. Kim , N.‐G. Park , Nat. Energy 2016, 1, 16081.

[33] Q. Chen , H. Zhou , T. B. Song , S. Luo , Z. Hong , H. S. Duan , L. Dou , Y. Liu , Y. Yang , Nano Lett. 2014, 14, 4158. 24960309 10.1021/nl501838y

[34] M. I. Saidaminov , A. L. Abdelhady , B. Murali , E. Alarousu , V. M. Burlakov , W. Peng , I. Dursun , L. Wang , Y. He , G. Maculan , A. Goriely , T. Wu , O. F. Mohammed , O. M. Bakr , Nat. Commun. 2015, 6, 7586. 26145157 10.1038/ncomms8586 PMC4544059

[35] Z. Chen , Q. Dong , Y. Liu , C. Bao , Y. Fang , Y. Lin , S. Tang , Q. Wang , X. Xiao , Y. Bai , Y. Deng , J. Huang , Nat. Commun. 2017, 8, 1890. 29192232 10.1038/s41467-017-02039-5 PMC5709415

[36] P. G. Harrison , A. T. Steel , J. Organomet. Chem. 1982, 239, 105.

[37] Z. Zeng , J. Zhang , X. Gan , H. Sun , M. Shang , D. Hou , C. Lu , R. Chen , Y. Zhu , L. Han , Adv. Energy Mater. 2018, 8, 1801050.

[38] N. Ahn , D. Y. Son , I. H. Jang , S. M. Kang , M. Choi , N. G. Park , J. Am. Chem. Soc. 2015, 137, 8696. 26125203 10.1021/jacs.5b04930

[39] F. Huang , Y. Dkhissi , W. Huang , M. Xiao , I. Benesperi , S. Rubanov , Y. Zhu , X. Lin , L. Jiang , Y. Zhou , A. Gray‐Weale , J. Etheridge , C. R. McNeill , R. A. Caruso , U. Bach , L. Spiccia , Y.‐B. Cheng , Nano Energy 2014, 10, 10.

[40] F. Li , W. Zhu , C. Bao , T. Yu , Y. Wang , X. Zhou , Z. Zou , Chem. Commun. 2016, 52, 5394. 10.1039/c6cc00753h 27009444

[41] J. Luo , J. Xia , H. Yang , H. A. Malik , F. Han , H. Shu , X. Yao , Z. Wan , C. Jia , Nano Energy 2020, 70, 104509.

[42] Q. Jiang , Z. Chu , P. Wang , X. Yang , H. Liu , Y. Wang , Z. Yin , J. Wu , X. Zhang , J. You , Adv. Mater. 2017, 29, 1703852. 10.1002/adma.201703852 29044741

[43] D. Luo , W. Yang , Z. Wang , A. Sadhanala , Q. Hu , R. Su , R. Shivanna , G. F. Trindade , J. F. Watts , Z. Xu , T. Liu , K. Chen , F. Ye , P. Wu , L. Zhao , J. Wu , Y. Tu , Y. Zhang , X. Yang , W. Zhang , R. H. Friend , Q. Gong , H. J. Snaith , R. Zhu , Science 2018, 360, 1442. 29954975 10.1126/science.aap9282

[44] L. Wang , C. McCleese , A. Kovalsky , Y. Zhao , C. Burda , J. Am. Chem. Soc. 2014, 136, 12205. 25145978 10.1021/ja504632z

[45] J. Xia , J. Luo , H. Yang , F. Zhao , Z. Wan , H. A. Malik , Y. Shi , K. Han , X. Yao , C. Jia , Adv. Funct. Mater. 2020, 30, 2001418.

[46] Z. Liu , J. Hu , H. Jiao , L. Li , G. Zheng , Y. Chen , Y. Huang , Q. Zhang , C. Shen , Q. Chen , H. Zhou , Adv. Mater. 2017, 29, 1606774. 10.1002/adma.201606774 28417481

[47] X. Li , M. J. Frisch , J. Chem. Theory Comput. 2006, 2, 835. 26626690 10.1021/ct050275a

[48] Q. Jiang , D. Rebollar , J. Gong , E. L. Piacentino , C. Zheng , T. Xu , Angew. Chem., Int. Ed. 2015, 54, 7617. 10.1002/anie.201503038 25968343

[49] J.‐Y. Seo , T. Matsui , J. Luo , J.‐P. Correa‐Baena , F. Giordano , M. Saliba , K. Schenk , A. Ummadisingu , K. Domanski , M. Hadadian , A. Hagfeldt , S. M. Zakeeruddin , U. Steiner , M. Grätzel , A. Abate , Adv. Energy Mater. 2016, 6, 1600767.

[50] F. M. Li , C. T. Zhu , S. Y. Ma , A. M. Sun , H. S. Song , X. B. Li , X. Wang , Mater. Sci. Semicond. Process. 2013, 16, 1079.

[51] S. C. Liu , Z. Li , Y. Yang , X. Wang , Y. X. Chen , D. J. Xue , J. S. Hu , J. Am. Chem. Soc. 2019, 141, 18075. 31638806 10.1021/jacs.9b07182

[52] P. Zeng , G. Feng , X. Cui , M. Liu , J. Phys. Chem. C 2020, 124, 6290.

[53] M. Hu , C. Bi , Y. Yuan , Z. Xiao , Q. Dong , Y. Shao , J. Huang , Small 2015, 11, 2164. 25641931 10.1002/smll.201402905

[54] Y. Wu , F. Xie , H. Chen , X. Yang , H. Su , M. Cai , Z. Zhou , T. Noda , L. Han , Adv. Mater. 2017, 29, 1701073. 10.1002/adma.201701073 28524262

[55] S. Bai , P. Da , C. Li , Z. Wang , Z. Yuan , F. Fu , M. Kawecki , X. Liu , N. Sakai , J. T. Wang , S. Huettner , S. Buecheler , M. Fahlman , F. Gao , H. J. Snaith , Nature 2019, 571, 245. 31292555 10.1038/s41586-019-1357-2

[56] M. Kim , G.‐H. Kim , T. K. Lee , I. W. Choi , H. W. Choi , Y. Jo , Y. J. Yoon , J. W. Kim , J. Lee , D. Huh , H. Lee , S. K. Kwak , J. Y. Kim , D. S. Kim , Joule 2019, 3, 2179.

[57] A. Mei , X. Li , L. Liu , Z. Ku , T. Liu , Y. Rong , M. Xu , M. Hu , J. Chen , Y. Yang , M. Gratzel , H. Han , Science 2014, 345, 295. 25035487 10.1126/science.1254763

