# Supplementary material for: Morphology Tuning and Its Role in Optimization of Perovskite Films Fabricated from A Novel Nonhalide Lead Source
Source: Adv Sci (Weinh). 2020 Nov 1;7(23):2002296. doi: 10.1002/advs.202002296 (PMC7709991; doi:10.1002/advs.202002296)
Supplement: Supplementary file 1 — Supporting information [file ADVS-7-2002296-s001.pdf]

© 2020 Wiley-VCH GmbH

ADVANCED SCIENCE Open Access

Supporting Information

for *Adv. Sci.*, DOI: 10.1002/advs.202002296

Morphology Tuning and Its Role in Optimization of Perovskite Films Fabricated from A Novel Nonhalide Lead Source

*Jinwen Gu, Faming Li, Zenghui Wang, Yiran Xie, Lihe Yan, Peng Zeng, Hua Yu, and Mingzhen Liu**

Supporting Information

Morphology Tuning and Its Role in Optimization of Perovskite Films Fabricated from A Novel Non-halide Lead Source

*Jinwen Gu[†], Faming Li[†], Zenghui Wang, Yiran Xie, Lihe Yan, Peng Zeng, Hua Yu, Mingzhen Liu**

J. Gu, Dr. F. Li, Y. Xie, Dr. P. Zeng, Prof. M. Liu
School of Materials and Energy, University of Electronic Science and Technology of China, Chengdu 611731, P.R. China.
Email: mingzhen.liu@uestc.edu.cn

J. Gu, Dr. F. Li, Prof. Z. Wang, Y. Xie, Dr. P. Zeng, Prof. M. Liu
Center for Applied Chemistry, University of Electronic Science and Technology of China, Chengdu 611731, P.R. China.
Email: mingzhen.liu@uestc.edu.cn

Prof. L. Yan
Key Laboratory for Physical Electronics and Devices of the Ministry of Education & Shaanxi Key Lab of Information Photonic Technique, School of Electronics & information Engineering, Xi'an Jiaotong University, Xi'an 710049, P.R. China.

Prof. H. Yu
Institute of Photovoltaics, Southwest Petroleum University, Chengdu 610500, P.R. China.

[†] These authors contributed equally to this work.

Experimental section

Materials: Lead (II) formate ($Pb(HCOO)_2$, >90%, Alfa Aesar), Lead acetate ($Pb(CH_3COO)_2$, 99%, Aladdin Industrial Corporation), Lead(II) iodide (PbI_2, 99.9%, Sigma-Aldrich), Methylammonium iodide (MAI, Dyesol), Methylammonium chlorine (MACl, Dyesol), Tin(IV) oxide colloid precursor ((SnO_2, 15% in H_2O colloidal dispersion, Alfa Aesar), TiO_2 paste (Dyesol 18NR-T), N,N-dimethylformamide (DMF, 99.8%, Sigma–Aldrich), Dimethyl sulfoxide (DMSO, 99.8%, Sigma–Aldrich), Chlorobenzene (99.8%, Sigma–Aldrich), Bis(trifluoromethane) sulfonamide lithium salt (Li-TFSI, 99.95%, Sigma–Aldrich), 4-tert-Butylpyridine (*t*BP, 96%, Sigma–Aldrich), Spiro-OMeTAD (99.8%, Borun New Material Technology). All reagents and solvents were used without further purification.

Preparation of $MAPbI_3$ films via the $Pb(HCOO)_2$ route. The precursor solution for $MAPbI_3$ was prepared by dissolving $Pb(HCOO)_2$ and MAI in DMF solution with different molar ratios (1:3.00, 1:3.15, 1:3.30, and 1:3.45; the molar amount of Pb^{2+} is fixed at 1.1mol/L), followed by stirring the solution at 80°C for 20min. To increase solubility of lead source and control the crystal growth rate, we added DMSO with a high boiling point (189°C) to the $Pb(HCOO)_2$/MAI/DMF precursor. The concentrations of DMSO additive was investigated at 0%, 5%, 10%, 15%, the optimal processing condition involves applying 10% DMSO additive was determined. Then a hydrophilic polytetrafluoroethylene (PTFE) syringe filter (pore size of 0.22μm) was adopted to purify the as-prepared precursor solution, 70μL of which was spin-coated onto the substrate (glass/FTO/compact TiO_2/mesoporous TiO_2 or glass/FTO/compact SnO_2) at 2800rpm for 40s. During the spinning process, about 58psi continuous nitrogen gas (room temperature) stream was blown over the film till the spin-coating finishes. At last, the substrates were annealed at 100°C on a hot plate for 20min. All of the procedures were carried out in a glovebox under the atmosphere of nitrogen.

Further, in order to improve photoelectric characteristics of solar cells, MACl (0mg/mL, 7mg/mL, 14mg/mL, 21mg/mL) was added into perovskite precursor solution with a $Pb(HCOO)_2$:MAI molar ratio of 1:3.15, the champion device was obtained at a concentration of 14mg/mL. The perovskite films based on with/without additive of MACl were fabricated through the similar procedure.

Preparation of $MAPbI_3$ films via the $Pb(CH_3COO)_2$ route. The $MAPbI_3$ perovskite films were prepared using $Pb(CH_3COO)_2$ source, by varying the ratios of $Pb(CH_3COO)_2$ and MAI in DMF with DMSO additives (See Supplemental Information for more information). Then the precursor solution was spin-coated on the glass substrate at 2800rpm for 40s. During the spinning process, about 58 psi continuous

nitrogen gas (room temperature) stream was blown over the film until the spin-coating finished. At last, the $MAPbI_3$ perovskite films using $Pb(CH_3COO)_2$ source were annealed at 100°C for 15min (optimized conditions). These $MAPbI_3$ films were used for characterize their surface morphology and carrier lifetime.

Device fabrication. Glass substrates with fluorine-doped tin-oxide (FTO) coating were cleaned with three different solvents in a sequence of deionized water, acetone and ethanol, followed by being purged in a plasma chamber for 8min. The compact SnO_2 layer was formed via spin-coating diluted SnO_2 colloid precursor (diluted in water, v/v 1:3) onto the pre-cleaned FTO glass substrates, at 3000rpm for 30s and then annealed at 150°C for 30min. The perovskite films were deposited on top of the substrate (glass/FTO/compact SnO_2 or glass/FTO/compact TiO_2/mesoporous TiO_2). The HTM layer was prepared by dissolving 2,2′,7,7′-tetrakis(N,N-dip-methoxyphenylamine)-9,9′-spirobifluorene (spiro-OMeTAD), lithium Li-TFSI and 4-tert-butylpyridine (4-*t*BP) in chlorobenzene and spin-coating the solution onto the perovskite layer. At last, an Au layer with a thickness of 100nm was coated to form metal electrodes via thermal evaporation.

Characterization. X-ray diffraction spectra were obtained with X-ray diffraction (Bruker D8-Advance, Cu K_α radiation). The field-emission SEM (Hitachi S-4300) was employed to acquire SEM images, the electron beam was accelerated at 10kV. Thermogravimetry analysis was conducted by TG-DTG (NETZSCH, STA-449-C) in the nitrogen atmosphere. UV-vis absorption measurements were measured at room temperature through UV-vis spectrophotometer (PerkinElmer Lamuda365). Femtosecond transient absorption spectroscopy was conducted by using an amplified Ti:Sapphire laser source (Coherent Legend, 800nm, 100fs, 2mJ per pulse, and 1kHz repetition rate). The laser output was split into two parts; the stronger beam was frequency doubled to generate 400nm excitation light, and the other one was focused into a 1mm thick sapphire plate to generate broad-band white-light probe pulses. The TA data were detected by a spectrometer at various time delays between the pump and probe pulses. The cross-sectional TEM image was prepared in a focus ion beam system (FEI HELIOS NanoLab 600i FIB). The surface roughness and phase were characterized by an AFM (KEYSIGHT Technologies 7500) with a Pt-coated conductive cantilever probe (Bruker, Model: SCM-PIT-V2) for KPFM technique. Fourier transform infrared spectroscopy (FTIR) spectra were collected by a Perkin-Elmer Spectrum GX FTIR spectrometer. Steady-state PL and time-resolved PL measurements were conducted on a time correlated single photon counting (TCSPC) system (FluoTime 300, PicoQuant). The TRPL decay profiles were recorded at 768nm

for all of the samples with a probing width of 5nm upon excitation by a 510nm laser (LDH-P-C-510, PicoQuant GmbH). X-ray Photoelectron Spectroscopy (XPS) was used to examine the presence of oxygen inside films etched by Ar^+ plasma (ThermoFisher ESCALAB 250Xi). The vacuum degree of the analysis chamber is about 5×10^{-9} mbar. The space-charge-limited current (SCLC) measurements were carried out by a Keithley 2400 digital source-meter under dark condition. The J-V curves of the PSCs devices were measured using the Keithley 2400 series digital source-meter unit under simulated AM 1.5G irradiation (100mW cm^{-2}, xenon-lamp, Newport). The light intensity was calibrated by the standard reference of a Newport Si solar cell before measurements. The mask with a square aperture (0.09 cm^2) close to the top of the PSCs device was used to define the effective area. The J-V curves were measured from forward bias to reverse bias; the scan rate was 100mV/s. External quantum efficiency (EQE) was measured using a measurement system (model QE-R, Enli Technology Co., Ltd.). The devices testing were carried out in ambient air.

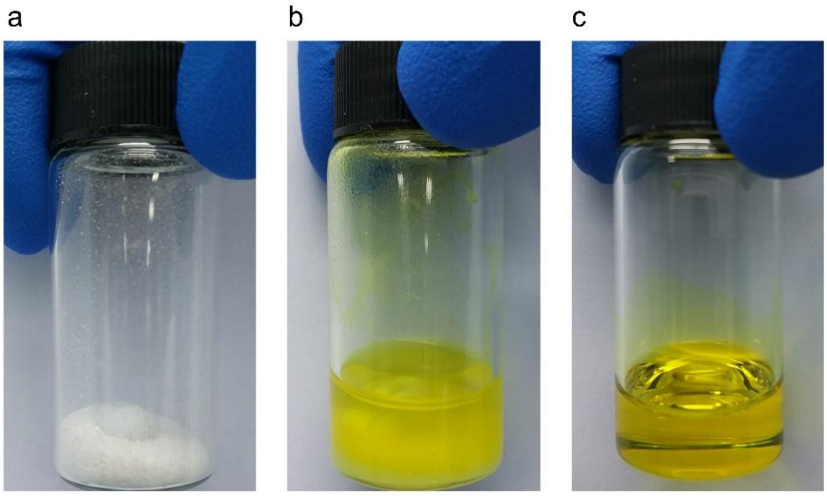

Figure S1. Optical images of $Pb(HCOO)_2$ in different states. (a) Mixed solid powder of $Pb(HCOO)_2$ and MAI. (b) $Pb(HCOO)_2$ and MAI dissolved in DMF solvent with DMSO. (c) Above solution after heating and stirring for 20 min.

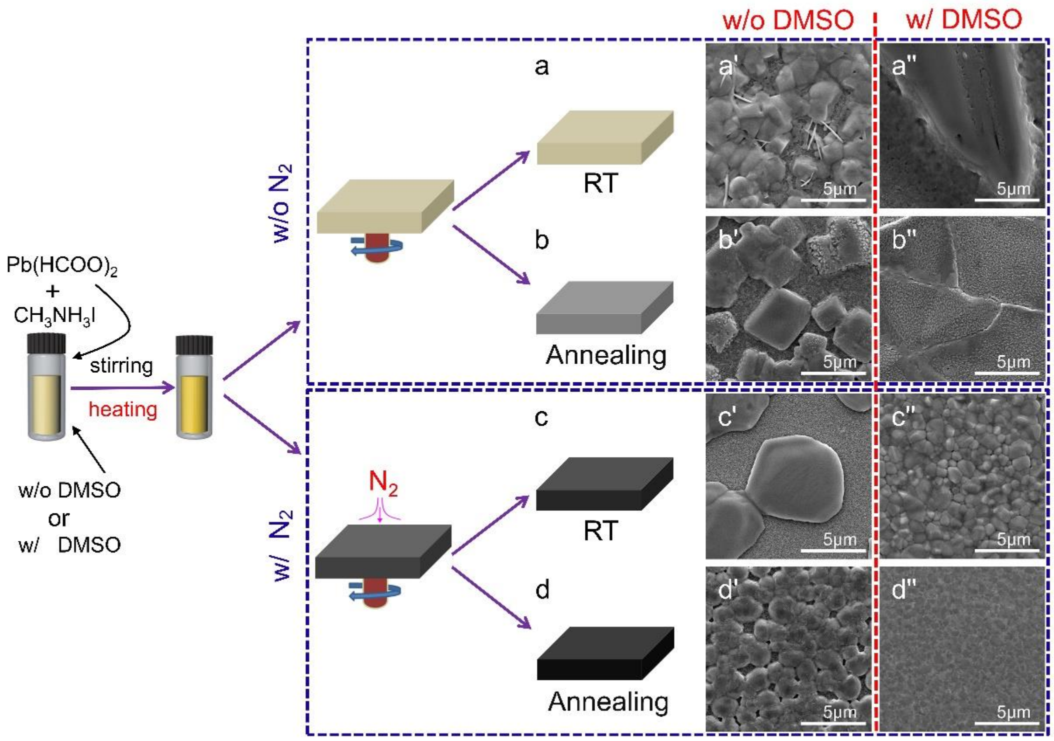

Figure S2. Fabrication procedure and top-morphology SEM images. $Pb(HCOO)_2$ and MAI are mixed in DMF with/without DMSO. After stirring and heating, the film was prepared from the solution under different conditions: (a) without N_2 flowing and annealing treatment; (b) without N_2 flowing but with annealing; (c) with N_2 flowing but without annealing; (d) with N_2 flowing and annealing; (a'-d') and (a"-d") represent SEM images for perovskite films without/with DMSO additive in precursors solution respectively.

Figure S2a'-d' and a"-d" show the scanning electron microscope (SEM) images of the resulting films from 8 different processing conditions, from which we make the following observations. First, introduction of nitrogen flow during spin-coating improves the uniformity and crystallization of the resulting film, due to the super-saturation of the perovskite components as a result of fast volatilization of solvent in the wet film. This allows one to obtain uniform perovskite films without using highly toxic anti-solvents such as chlorobenzene, toluene, diethyl ether, which were often involved in lead halide routes.[1,2] Second, adding DMSO offers greater control of the crystal growth rate through improved solubility of the lead source,[1] resulting in high-quality perovskite films free of pin-holes (Figure S2a"-d"). Third, post-annealing improves the film coverage, as the elevated temperature provides additional thermal energy to assist the crystallization of the perovskite material, and further enhances the efficiency of solvent removal (Figure S2d").

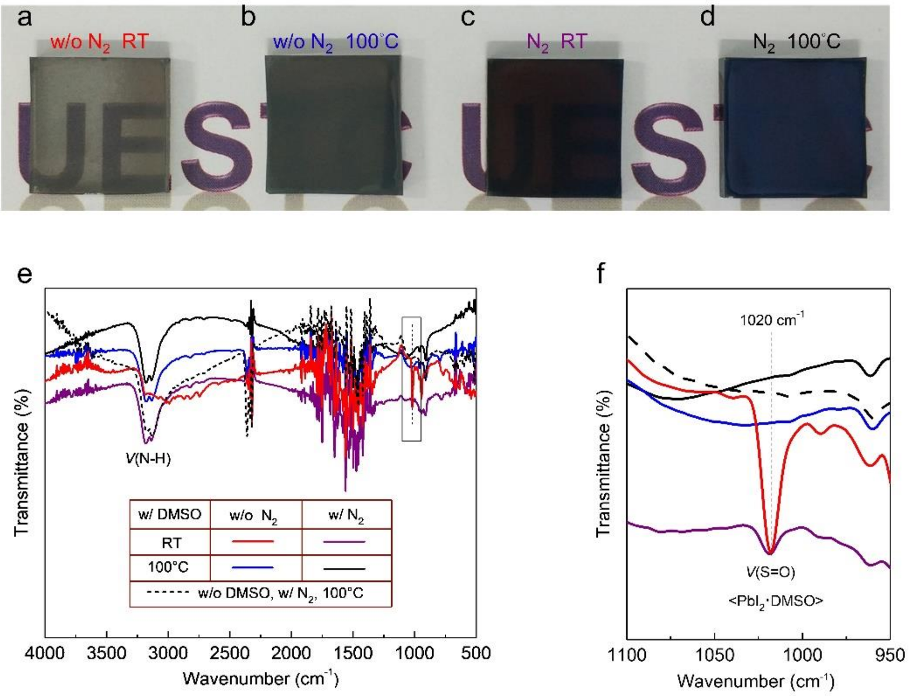

Figure S3. Analysis of crystalline state. The photo images of perovskite films under four different conditions (the size of samples is 20 × 20 mm^2): (a) without N_2 flow nor annealing; (b) without N_2 flow but with annealing; (c) with N_2 flow but without annealing; (d) with N_2 flow and annealing; (e) The FTIR of the perovskite films treated under the four corresponding different conditions; (f) zoom-in of the region around the S=O vibrations.

We further characterized the composition of the resulting films using Fourier transform infrared spectroscopy (FTIR), which provides information about the vibrational states of the molecules. (Figure S3e) shows the FTIR data of samples in (Figure S3 a-d) along with corresponding optical images. Films with nitrogen or thermal treatments exhibit dark colour (Figure S3 b-d), consistent with the two IR peaks around 3200~3450 cm^{-1} corresponding to the N-H stretching vibration (Figure S3e), suggesting formation of perovskite phase.[2] In contrast, samples without these treatments show yellowish colour (Figure S3a) and no such IR peaks, indicating absence of perovskite phase. Furthermore, we found that the S=O vibration peak at 1020 cm^{-1} (Figure S3f), indicative of PbI_2·DMSO adduct and thus incomplete reaction,[1] can be significantly reduced by flowing nitrogen, and completely eliminated after annealing. As control, we also show data for sample without DMSO additive (dashed lines in Figure S3 e&f).

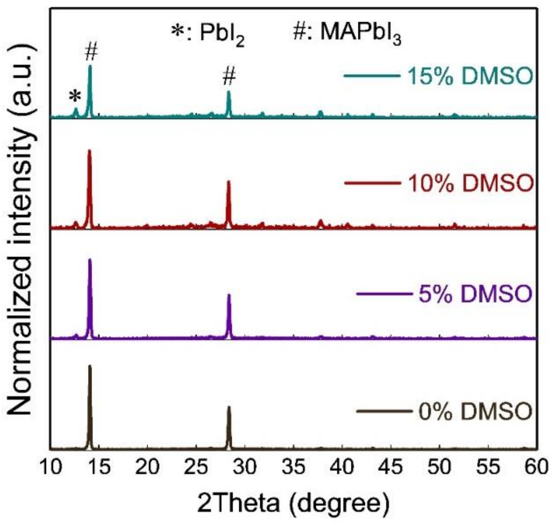

Figure S4. XRD patterns of perovskite films via $Pb(HCOO)_2$ route, with various ratios of DMSO in the precursor solution (The characteristic peaks of PbI_2 and $MAPbI_3$ are marked with asterisk (*) and pound (#) signs).

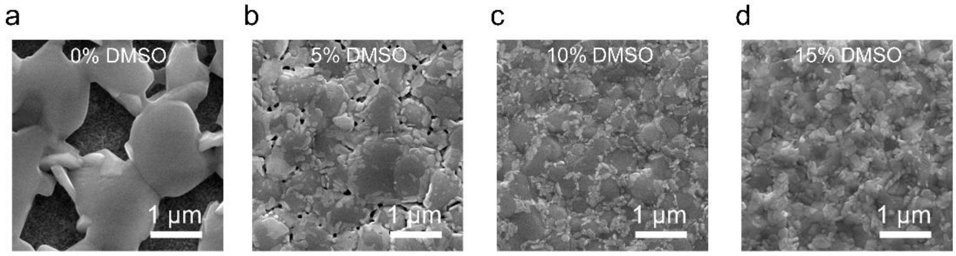

Figure S5. Top-view SEM images of perovskite films via $Pb(HCOO)_2$ route, with various ratios of DMSO in the precursor solution.

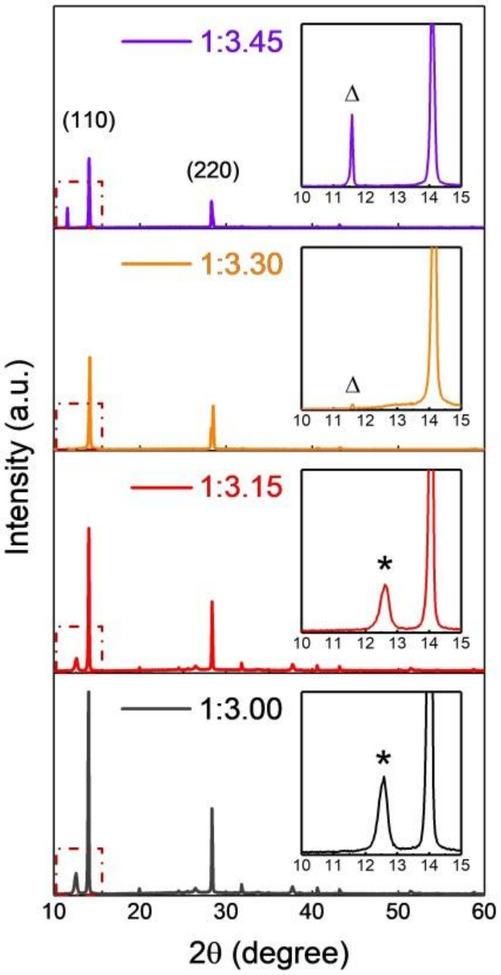

Figure S6. XRD patterns of $MAPbI_3$ perovskite films with various ratios of $Pb(HCOO)_2$/MAI in the precursors. Insets show zoomed-in XRD spectra, the characteristic peaks of PbI_2 and MAI are marked with asterisk (*) and triangle (△), respectively.

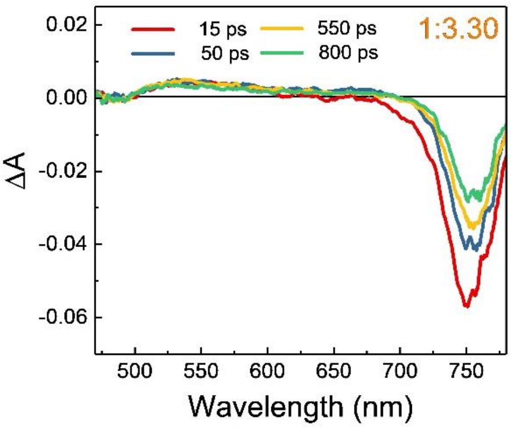

Figure S7. fs-TA spectra of perovskite films at various delays after photoexcitation, the perovskite films with the ratio of $Pb(HCOO)_2$: MAI = 1:3.30.

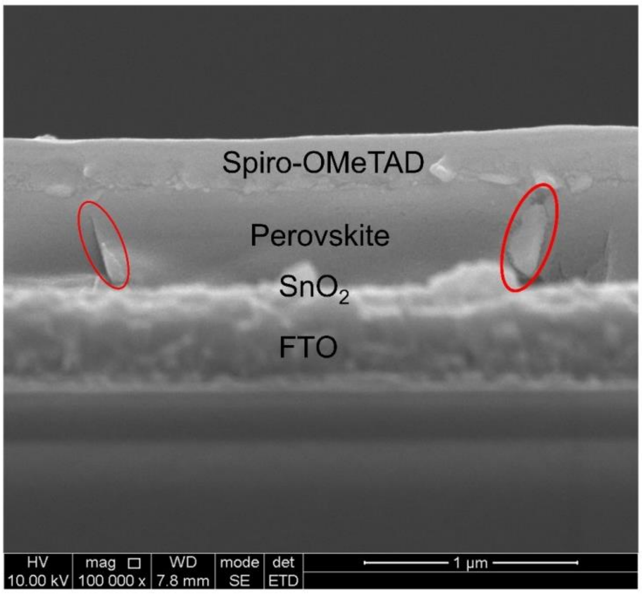

Figure S8. The cross-sectional SEM image of PSCs (Bright PbI_2 are labeled with red circles). The perovskite films based on the MACl additive (14mg/mL) were fabricated through the same procedure.

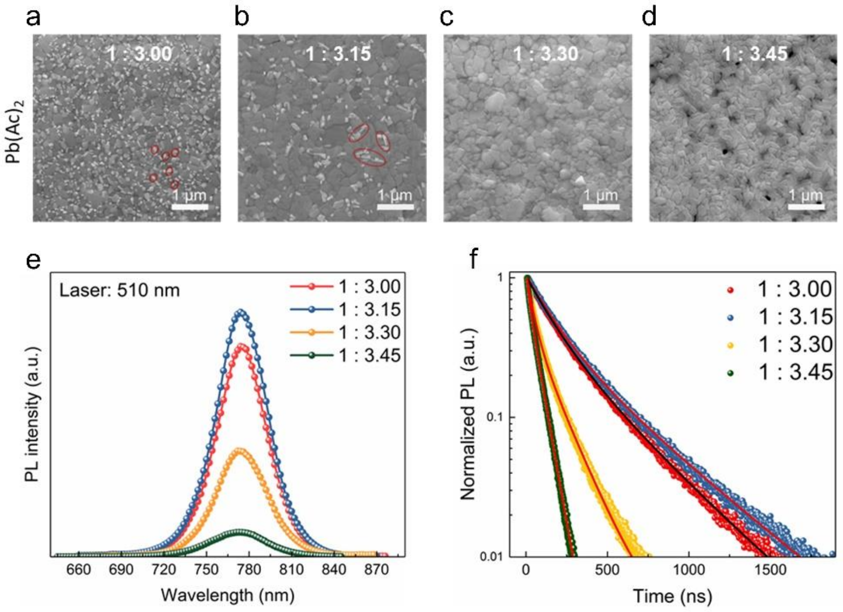

Figure S9. Morphology, PL and TRPL of the perovskite films via $Pb(Ac)_2$ routes. (a-d) Top-view SEM images, (e) PL and (f)TRPL decays curves of perovskite films fabricated from the different molar ratios of $Pb(Ac)_2$ and MAI.

For better understanding the spontaneous formation of PbI_2 at GBs for such non-halide source, we also fabricate the perovskite film from another similar acid-based lead source, lead acetate ($Pb(CH_3COO)_2$, $Pb(Ac)_2$) via the same route for comparison. As expected, small crystals also show up at GBs of perovskite films (Figure S9 a-b). However, the distribution of these small crystals at GBs shows great difference between non-halide lead sources $Pb(HCOO)_2$ and $Pb(Ac)_2$ (Figure 2 a-b and Figure S9 a-b). The small crystals in white colours tightly and orderly wrap around the GBs of the perovskite via $Pb(HCOO)_2$ route while it intersperses loosely at GB of perovskite via $Pb(Ac)_2$ source. We suspect that the difference in crystal distribution is the result of non-halide anions induction，previous studies also indicated that some lead anions such as CH_3COO^-, Cl^- and SCN^- have a special contribution to crystal growth and film morphology.[3,4,5] For $Pb(HCOO)_2$ route, the intermediate of MACOOH as an ionic liquid has an extremely low vapor pressure,[6] it remains in the perovskite film and retards the rate of crystal growth after the solvent evaporated, but the similar ionic liquid cannot be formed spontaneously in $Pb(Ac)_2$ route. Therefore, excess cations of lead salts are evenly squeezed to GBs rather than dispersedly agglomerated.

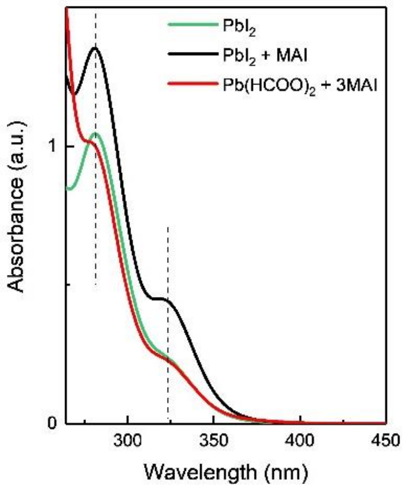

Figure S10. Absorption spectra of precursor solution with PbI_2, PbI_2+MAI, $Pb(HCOO)_2$+3MAI in DMF.

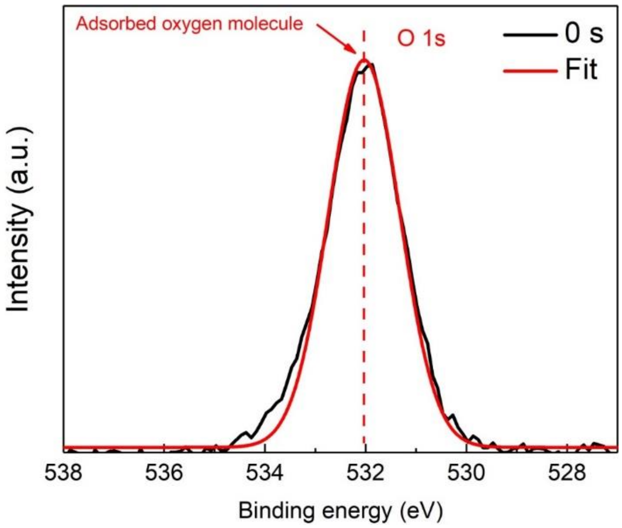

Figure S11. XPS of the O 1s of surface perovskite film with the ratio of $Pb(HCOO)_2$: MAI = 1:3.15.

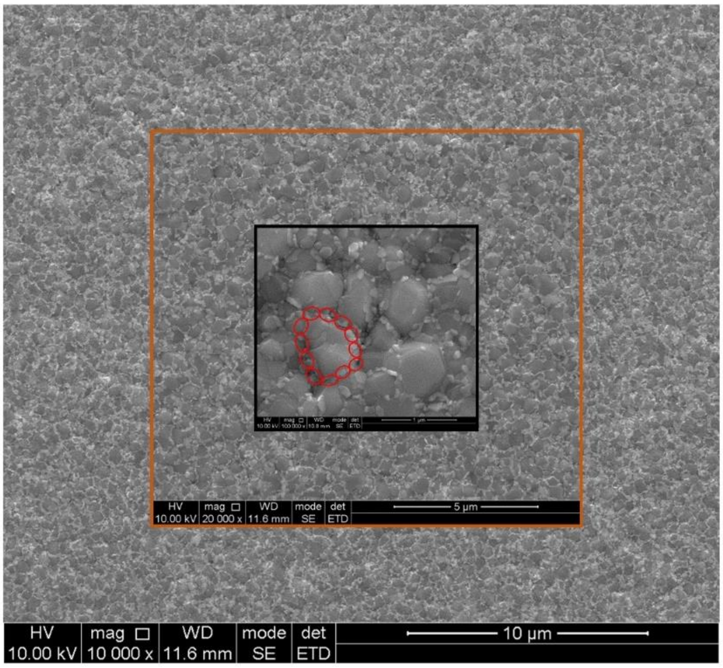

Figure S12. The different magnification top morphology of perovskite film (Bright PbI_2 are labeled with red circles).

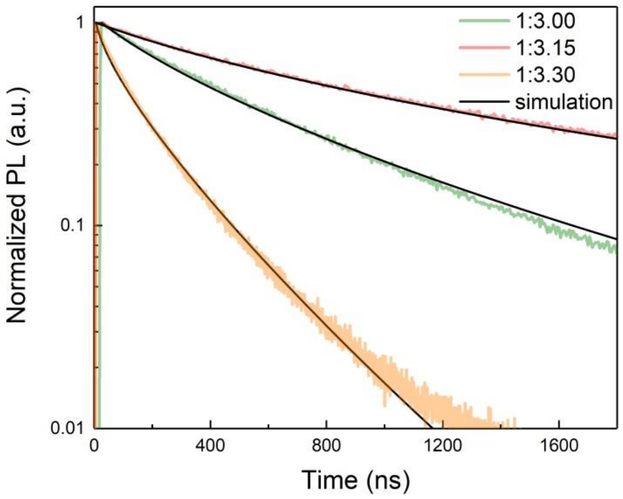

Figure S13. Comparison of PL decay simulation results with contributions from radiative recombination at surface and bulk.

We applied the charge carrier recombination-diffusion model involving surface recombination velocities (SRVs) to fit the PL decay measurement results, based on our previous work[29]. Especially, the SRVs reflect the defect density at grain surfaces. With no consideration on auger recombination process, the recombination-diffusion model is applied as:

$$\frac{\partial n(x,t)}{\partial t} = D\frac{\partial^2 n}{\partial x^2} - k_1 n - k_2 n^2$$

where n is the carrier density, D the diffusion constant, k_1 and k_2 correspond to the widely known monomolecular and germinate recombination rates, respectively. Initial and boundary conditions are given as:

$$n(x,0) = n(0,0) \times e^{-ax}$$

$$\frac{\partial n(x,t)}{\partial t}|_{x=0} = \frac{S_0}{D} n(0,t)$$

$$\frac{\partial n(x,t)}{\partial t}|_{x=L} = -\frac{S_L}{D} n(L,t)$$

In which S_0 and S_L refers to the SRV at both surfaces of the film, respectively. More information can be found in this article.[29]

Based on the above model and the experimental PL decays, the simulation results are shown in Figure A1. With the listed parameters in Table S3, the simulation results are in good agreement with the measurements.

We can clearly find that the 1:3.15 sample (τ_2=1714ns) possessed significantly reduced defects (S_0 and S_L) compared to the 1:3.30 sample (τ_2=264ns). The monomolecular recombination rate k_1 also shows a reduction in the 1:3.15 sample. We suggest that k_1 is associated with the trap-assisted recombination, thus the reduction in k_1 also verified the reduced defect density in the 1:3.15 sample.

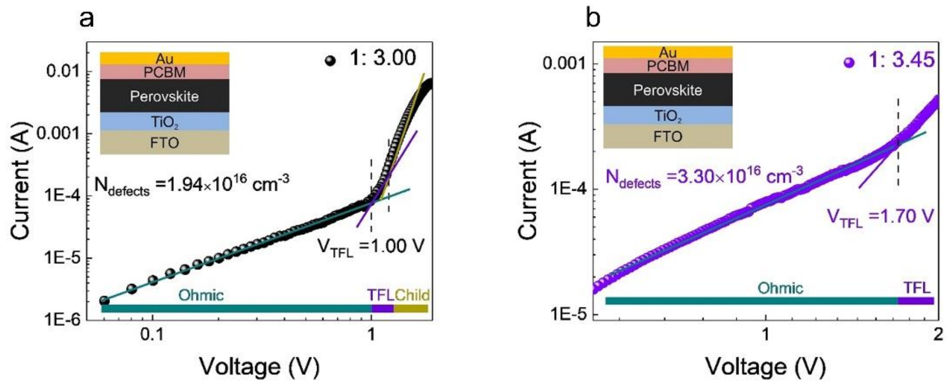

Figure S14. Current-voltage curve for an electron-only device (structure: Au/PCBM/Perovksite /TiO_2/FTO/Glass), the perovskite films based on the ratio of $Pb(HCOO)_2$: MAI=1:3.00 (a), and 1:3.45 (b).

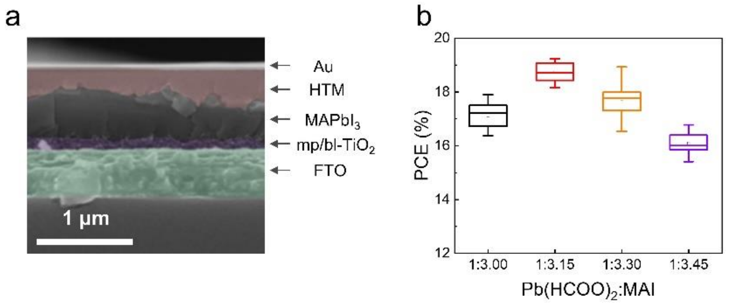

Figure S15. (a) The cross-sectional SEM image of PSCs, with a device structure of glass/FTO/TiO_2 compact layer/TiO_2 mesoporous layer/perovskite/HTM/Au. (b) The different Molar ratios of $Pb(HCOO)_2$:MAI from the statistical distribution of the device performance for 20 devices.

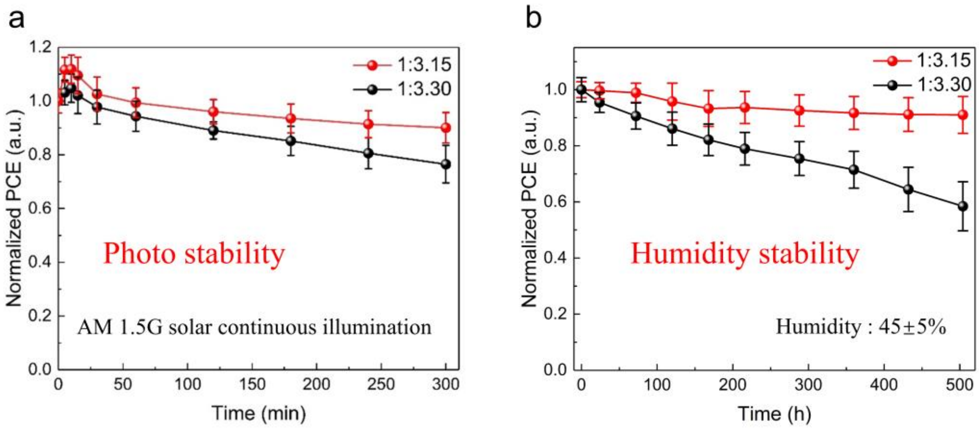

Figure S16. (a) The photo stability of the devices (molar ratio of $Pb(HCOO)_2$:MAI =1: 3.15 and 1: 3.30) was conducted under operational conditions in a glove box with N_2 atmosphere, (b) the humidity stability of these devices were tested by exposing them to an environmental atmosphere with 45±5% RH, and their J-V curves were collected constantly.

Table S1. The statistical table of nonlinear fitting for time-resolved PL decays of perovskite films (by $Pb(HCOO)_2$ routes with different ratio of MAI) on bare glass.

Samples	A_1 (%)	τ_1 (ns)	A_2 (%)	τ_2 (ns)	τ_{avg} (ns)
3.00	50.0%	337	50.0%	921	629
3.15	39.0%	460	61.0%	1714	1224
3.30	45.2%	82	54.8%	264	182
3.45	85.4%	8	14.6%	104	22

Table S2. The statistical table of nonlinear fitting for time-resolved PL decays of perovskite films (by $Pb(Ac)_2$ routes with different ratio of MAI) on bare glass.

Samples	A_1 (%)	τ_1 (ns)	A_2 (%)	τ_2 (ns)	τ_{avg} (ns)
3.00	57.0%	150	43.0%	384	250
3.15	57.8%	155	42.2%	437	274
3.30	57.3%	43	42.7%	157	91
3.45	29.2%	15	70.8%	64	58

Table S3. List of simulation results of perovskite/glass samples.

$Pb(HCOO)_2$:MAI	1:3.00	1:3.15	1:3.30
D [cm^2/s]	6×10^{-5}	6×10^{-5}	6×10^{-5}
k_1 [s^{-1}]	3×10^{5}	1×10^{5}	1.3×10^{6}
k_2 [cm^3/s]	1×10^{-10}	1×10^{-10}	1×10^{-10}
S_0 [cm/s]	5	0	100
S_L [cm/s]	0	0	0

Table S4. The PCE of perovskite solar cells by pure non-halide lead.

Lead source	Perovskite films	PCE(%)	References
$Pb(Ac)_2$	$MAPbI_{1-x}Br_x$	18.72	[7]
$Pb(Ac)_2$	$MAPbI_{1-x}Br_x$	18.32	[8]
$Pb(Ac)_2$	$MAPbI_3$	18.50	[9]
$Pb(Ac)_2$	$MAPbI_{1-x}Br_x$	16.33	[10]
$Pb(Ac)_2$	$MAPbI_3$	14.50	[11]
$Pb(Ac)_2$	$MAPbI_3$	17.96	[12]
$Pb(Ac)_2$	$MAPbI_3$	11.60	[13]
$Pb(Ac)_2$	$MAPbI_3$	15.20	[3]
$Pb(Ac)_2$	$MAPbI_3$	16.20	[14]
$Pb(Ac)_2$	$MAPbI_3$	10.80	[15]
$Pb(Ac)_2$	$MAPbI_3$	12.50	[16]
$Pb(SCN)_2$	$CH_3NH_3Pb(SCN)_2I$	8.30	[17]
$Pb(SCN)_2$	$MAPbI_{1-x}(SCN)_x$	>15.00	[18]
$Pb(SCN)_2$	$MAPbI_3$	7.60	[19]
$Pb(NO_3)_2$	$MAPbI_3$	12.58	[20]
$Pb(NO_3)_2$	$MAPbI_3$	8.40	[19]
$Pb(acac)_2$	$MAPbI_3$	1.50	[15]
**$Pb(HCOO)_2$**	**$MAPbI_3$**	**20.3**	**This work**

Table S5. The PCE of perovskite solar cells by mixing non-halide lead and halide lead.

Mixed lead source	Perovskite films	PCE(%)	References
$Pb(Ac)_2/PbI_2$ (6:4)	$MA_{0.6}FA_{0.4}PbI_3$	20.15	[21]
$Pb(SCN)_2/PbI_2$(5:95wt%)	$MAPbI_{3-x}(SCN)_x$	11.07	[22]
$Pb(Ac)_2/PbCl_2$ (9:1)	$MAPbI_3$	20.70	[23]
$Pb(SCN)_2/PbI_2$ (5:95wt%)	$MAPbI_3$	19.45	[24]
$Pb(SCN)_2/PbI_2$ (3:97%)	$MA_{0.7}FA_{0.3}PbI_3$	20.10	[25]
$Pb(Ac)_2/PbCl_2$ (1:1)	$MAPbI_3$	16.00	[26]
$Pb(Ac)_2/PbCl_2$ (6:1)	$MAPbI_3$	14.00	[27]
$Pb(Ac)_2/PbCl_2$ (4:1)	$MAPbI_3$	13.60	[28]

References

[1] N. Ahn, D. Y. Son, I. H. Jang, S. M. Kang, M. Choi and N. G. Park, *J. Am. Chem. Soc.*, 2015, **137,** 8696-8699.
[2] N. J. Jeon, J. H. Noh, Y. C. Kim, W. S. Yang, S. Ryu and S. I. Seok, *Nat. Mater.*, 2014, **13,** 897-903.
[3] W. Zhang, M. Saliba, D. T. Moore, S. K. Pathak, M. T. Horantner, T. Stergiopoulos, S. D. Stranks, G. E. Eperon, J. A. Alexander-Webber, A. Abate, A. Sadhanala, S. H. Yao, Y. L. Chen, R. H. Friend, L. A. Estroff, U. Wiesner and H. J. Snaith, *Nat. Commun.*, 2015, **6,** 6142.
[4] S. T. Williams, F. Zuo, C. C. Chueh, C. Y. Liao, P. W. Liang and A. K. Jen, *ACS Nano*, 2014, **8,** 10640-10654.
[5] D. T. Moore, H. Sai, K. W. Tan, D. M. Smilgies, W. Zhang, H. J. Snaith, U. Wiesner and L. A. Estroff,

J. Am. Chem. Soc., 2015, **137,** 2350-2358.
[6] J. Y. Seo, T. Matsui, J. S. Luo, J. P. Correa-Baena, F. Giordano, M. Saliba, K. Schenk, A. Ummadisingu, K. Domanski, M. Hadadian, A. Hagfeldt, S. M. Zakeeruddin, U. Steiner, M. Gratzel and A. Abate, *Adv. Energy Mater.*, 2016, **6,** 1600767.
[7] K. Chen, Q. Hu, T. Liu, L. Zhao, D. Luo, J. Wu, Y. Zhang, W. Zhang, F. Liu, T. P. Russell, R. Zhu and Q. Gong, *Adv. Mater.*, 2016, **28**, 10718-10724.
[8] L. Zhao, D. Luo, J. Wu, Q. Hu, W. Zhang, K. Chen, T. Liu, Y. Liu, Y. Zhang, F. Liu, T. P. Russell, H. J. Snaith, R. Zhu and Q. Gong, *Adv. Funct. Mater.*, 2016, **26,** 3508-3514.
[9] N. Giesbrecht, J. Schlipf, I. Grill, P. Rieder, V. Dyakonov, T. Bein, A. Hartschuh, P. Müller-Buschbaum and P. Docampo, *J. Mater. Chem. A*, 2018, **6,** 4822-4828.
[10] C. Li, Q. Guo, Z. Wang, Y. Bai, L. Liu, F. Wang, E. Zhou, T. Hayat, A. Alsaedi and Z. Tan, *ACS Appl. Mater. Interfaces*, 2017, **9,** 41937-41944.
[11] Y. Chen, A. Yerramilli, Y. Shen, Z. Zhao and T. Alford, *Solar Energy Materials and Solar Cells*, 2018, **174,** 478-484.
[12] T. Singh and T. Miyasaka, *Chem. Commun.*, 2016, **52,** 4784-4787.
[13] Q. Hu, L. Zhao, J. Wu, K. Gao, D. Luo, Y. Jiang, Z. Zhang, C. Zhu, E. Schaible, A. Hexemer, C. Wang, Y. Liu, W. Zhang, M. Gratzel, F. Liu, T. P. Russell, R. Zhu and Q. Gong, *Nat. Commun.*, 2017, **8,** 15688.
[14] W. Zhang, S. Pathak, N. Sakai, T. Stergiopoulos, P. K. Nayak, N. K. Noel, A. A. Haghighirad, V. M. Burlakov, D. W. deQuilettes, A. Sadhanala, W. Li, L. Wang, D. S. Ginger, R. H. Friend and H. J. Snaith, *Nat. Commun.*, 2015, **6,** 10030.
[15] F. K. Aldibaja, L. Badia, E. Mas-Marzá, R. S. Sánchez, E. M. Barea and I. Mora-Sero, *J. Mater. Chem. A*, 2015, **3,** 9194-9200.
[16] D. Forgács, M. Sessolo and H. J. Bolink, *J. Mater. Chem. A*, 2015, **3,** 14121-14125.
[17] Q. Jiang, D. Rebollar, J. Gong, E. L. Piacentino, C. Zheng and T. Xu, *Angew. Chem. Int. Ed.*, 2015, **54,** 1-5.
[18] Q. Tai, P. You, H. Sang, Z. Liu, C. Hu, H. L. Chan and F. Yan, *Nat. Commun.*, 2016, **7**, 11105.
[19] G. Balaji, P. H. Joshi, H. A. Abbas, L. Zhang, R. Kottokkaran, M. Samiee, M. Noack and V. L. Dalal, *Phys. Chem. Chem. Phys.*, 2015, **17**, 10369-10372.
[20] T. Y. Hsieh, T. C. Wei, K. L. Wu, M. Ikegami and T. Miyasaka, *Chem. Commun.*, 2015, **51,** 13294-13297.
[21] D. Luo, L. Zhao, J. Wu, Q. Hu, Y. Zhang, Z. Xu, Y. Liu, T. Liu, K. Chen, W. Yang, W. Zhang, R. Zhu and Q. Gong, *Adv. Mater.*, 2017, **29,** 1604758.
[22] Y. Chen, B. Li, W. Huang, D. Gao and Z. Liang, *Chem. Commun.*, 2015, **51,** 11997-11999.
[23] Z. Liu, L. Krückemeier, B. Krogmeier, B. Klingebiel, J. A. Márquez, S. Levcenko, S. Öz, S. Mathur, U. Rau, T. Unold and T. Kirchartz, *ACS Energy Lett.*, 2018, **4,** 110-117.
[24] W. Ke, C. Xiao, C. Wang, B. Saparov, H. S. Duan, D. Zhao, Z. Xiao, P. Schulz, S. P. Harvey, W. Liao, W. Meng, Y. Yu, A. J. Cimaroli, C. S. Jiang, K. Zhu, M. Al-Jassim, G. Fang, D. B. Mitzi and Y. Yan, *Adv. Mater.*, 2016, **28,** 5214-5221.
[25] C. Wang, D. Zhao, Y. Yu, N. Shrestha, C. R. Grice, W. Liao, A. J. Cimaroli, J. Chen, R. J. Ellingson, X. Zhao and Y. Yan, *Nano Energy*, 2017, **35,** 223-232.
[26] L. Ling, S. Yuan, P. Wang, H. Zhang, L. Tu, J. Wang, Y. Zhan and L. Zheng, *Adv. Funct. Mater.*, 2016, **26,** 5028-5034.
[27] J. Qing, H. T. Chandran, Y. H. Cheng, X. K. Liu, H. W. Li, S. W. Tsang, M. F. Lo and C. S. Lee, *ACS Appl. Mater. Interfaces*, 2015, **7,** 23110-23116.
[28] W. Qiu, T. Merckx, M. Jaysankar, C. Masse de la Huerta, L. Rakocevic, W. Zhang, U. W. Paetzold, R. Gehlhaar, L. Froyen, J. Poortmans, D. Cheyns, H. J. Snaith and P. Heremans, *Energy Environ. Sci.*, 2016, **9,** 484-489.
[29] P. Zeng, G. Feng, X. Cui, and M. Liu, *J. Phys. Chem. C* **2020**, *124*, 6290.
